# 3D-printed heart models for hands-on training in pediatric cardiology – the future of modern learning and teaching?

**DOI:** 10.3205/zma001544

**Published:** 2022-04-14

**Authors:** Barbara S. Brunner, Alisa Thierij, Andre Jakob, Anja Tengler, Maximilian Grab, Nikolaus Thierfelder, Christian J. Leuner, Nikolaus A. Haas, Carina Hopfner

**Affiliations:** 1 LMU Klinikum, Department of Pediatric Cardiology and Pediatric Intensive Care, Munich, Germany; 2 LMU Klinikum, Clinic and Polyclinic for Cardiac Surgery, Munich, Germany; 3 Etiopia-Witten e.V., Witten, Germany

**Keywords:** 3D-printed models, simulation training, medical education,, pediatric cardiology, congenital heart defects, diagnostic and interventional cardiac catheterizations

## Abstract

**Background:**

This project aims to develop a new concept in training pediatric cardiologists to meet the requirements of interventional cardiac catheterizations today in terms of complexity and importance. This newly developed hands-on training program is supposed to enable the acquisition of certain skills which are necessary when investigating and treating patients in a catheter laboratory.

**Methods:**

Based on anonymous CT-scans of pediatric patients’ digital 3D heart models with or without cardiac defects were developed and printed three-dimensionally in a flexible material visible under X-ray. Hands-on training courses were offered using models of a healthy heart and the most common congenital heart defects (CHD). An evaluation was performed by quantifying fluoroscopy times (FL-time) and a questionnaire.

**Results:**

The acceptance of theoretical and practical contents within the hands-on training was very positive. It was demonstrated that it is possible to master various steps of a diagnostic procedure and an intervention as well as to practice and repeat them independently which significantly reduced FL-time. The participants stated that the hands-on training led to more confidence in interventions on real patients.

**Conclusion:**

With the development of a training module using 3D-printed heart models, basic and advanced training in the field of diagnostic cardiac examinations as well as interventional therapies of CHD is possible. The learning effect for both, practical skills and theoretical understanding, was significant which underlines the importance of integrating such hands-on trainings on 3D heart models in education and practical training.

## Zusammenfassung

**Zielsetzung:**

Ziel dieses Projektes ist die Entwicklung eines neuen Aus- und Weiterbildungskonzepts in der Kinderkardiologie, um der zunehmenden Komplexität und dem Stellenwert interventioneller Eingriffe mittels Herzkatheter (HK) gerecht zu werden. Das neu entwickelte Hands-on Training soll den Erwerb entsprechender Fertigkeiten für die Durchführung der HK-Untersuchung an Patient*innen ermöglichen.

**Methodik:**

Basierend auf anonymisierten CT-Bildern von pädiatrischen Patient*innen wurden digitale 3D-Herzmodelle entwickelt und in einem flexiblen, unter Röntgenstrahlung sichtbaren Material dreidimensional ausgedruckt. Es fanden Hands-on Trainingskurse statt, bei denen Modelle eines gesunden Herzens sowie der häufigsten angeborenen Herzfehler (AHF) zum Einsatz kamen. Eine Evaluation erfolgte anhand der Quantifizierung von Durchleuchtungszeiten (DL-Zeit) und eines Fragebogens.

**Ergebnisse:**

Die Akzeptanz der theoretischen und praktischen Inhalte des Hands-on Trainings war sehr gut. Es hat sich gezeigt, dass sowohl das Erlernen diverser Schritte als auch selbstständiges Üben und Wiederholen und eine damit verbundene signifikante Verkürzung der benötigten DL-Zeit möglich sind. Die Teilnehmenden gaben an, dass das Hands-on Training zu mehr Sicherheit bei der Intervention an Patient*innen führen würde.

**Schlussfolgerung:**

Mit der Entwicklung eines Trainingsmoduls unter dem Einsatz 3D-gedruckter Herzmodelle sind Aus- und Weiterbildung im Bereich diagnostischer HK-Untersuchungen sowie interventioneller Therapien von AHF möglich. Der signifikante Lerneffekt sowohl für die praktischen Fähigkeiten als auch für das theoretische Verständnis spricht für die Integration des Simulationstrainings an 3D-Herzmodellen in Aus- und Weiterbildung.

## 1. Introduction

With a prevalence of 1% of all live births, congenital heart defects (CHDs) remain the most common congenital anomalies worldwide with treatment often indicated in early childhood [1]. While cardiac catheterizations used to be done mainly for diagnostic purposes nowadays there are also numerous therapeutic options to treat heart defects either completely or partially [2]. These interventional cardiac catheters (CC) replace open-heart surgery in many cases and, thus, offer a less invasive form of treatment with satisfying therapeutic outcomes and increasing patient safety at the same time [3]. To ensure optimal results in these highly complex interventions it is necessary to gain experience by appropriate and highly effective education and further training opportunities for pediatric cardiologists. This principle is already firmly implemented in other training areas such as aerospace. Regular simulation training in a standardized learning environment prepares both, young and experienced, pilots in the best possible way for various real-life (emergency) situations [4]. 

The high importance of constant repetition is also evident in emergency medicine, especially in resuscitation training [5]. Not only inexperienced but also experienced doctors consolidate their knowledge and practical skills through regular refresher courses. Therefore, regular standardized repetition of these training units is also recommended for experienced physicians and rescue workers and is mandatory in the Anglo-American regions [5]. 

Based on these considerations, a realistic simulation model for learning diagnostic CC-examinations was developed by our research group. In addition, the most frequent CHDs [6] were simulated using 3D-printed models to practice interventional CC. The aim of the study was to investigate whether a simulation training could be created promoting both, theoretical principles and practical skills, but also the understanding of the interventions by using the developed 3D printed models.

## 2. Project description

### 2.1. Model development 

**Figure 1:**
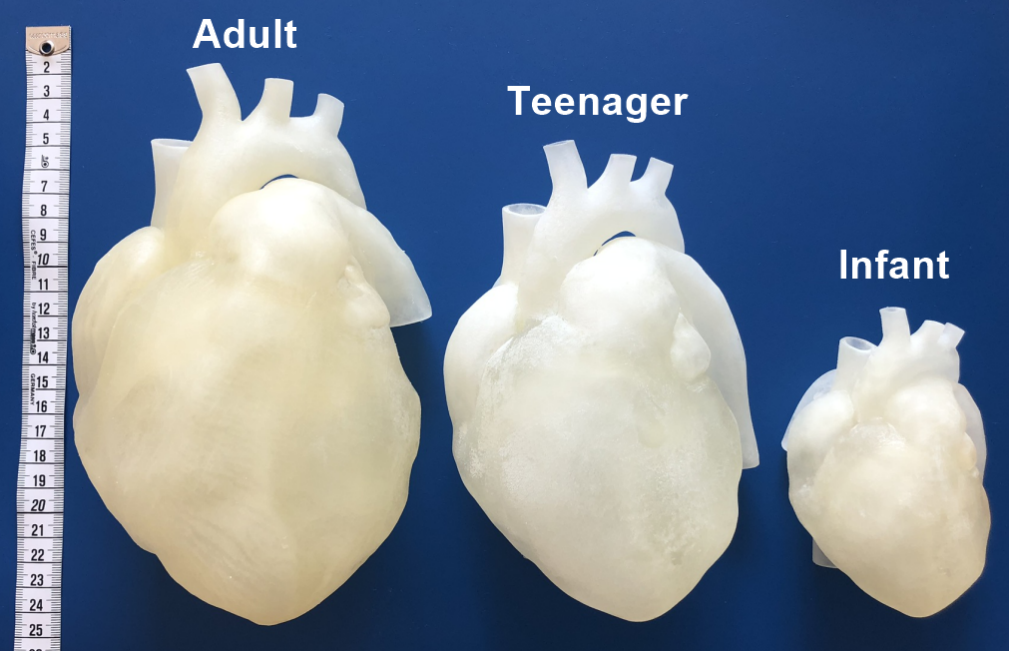
3D-printed heart models in different sizes The image shows 3D models of the heart in different sizes, i.e., of an adult, a teenager, and an infant. The heart models for the hands-on training were printed in an additive manufacturing process on a 3D-printer (Agilista 3200W, Keyence Corp.) using a flexible silicon rubber.

In several development steps the following models were continuously optimized: normal cardiovascular physiology and the most common CHDs: atrial septal defect (ASD), ventricular septal defect (VSD), persistent ductus arteriosus (PDA), aortic isthmus stenosis (ISTA) and aortic and pulmonary valve stenosis (AS, PS). The models used for the hands-on training were based on anonymized CT data of patients with CHD and children with healthy hearts which were obtained during clinically indicated examinations. Using a medical 3D software (Materialise Mimics Innovation Suite, Materialise NV), virtual 3D models of the intracardiac and intravascular volume were generated by segmenting the blood volume in the CT files. As a next step, an air-filled hollow model was created in each case so that the cardiac spaces in the 3D-printed models were accessible for training with catheters and wires via the hollow vessels. In addition, digital editing allowed to print an adult heart also in scaled sizes (adolescent heart 80%, neonatal heart 55%) (see figure 1 (Fig. 1)). For 3D printing on an Agilista 3200W Polyjet 3D printer (Keyence Corp.), a soft silicone rubber (AR-G1L, Keyence Corp.) was used, which was printed together with a water-soluble support material (AR-S1, Keyence Corp.) [https://www.keyence.de/products/3d-printers/3d-printers/agilista-3100/models/agilista-3200w/?search_sl=1]. The process of 3D model creation from data acquisition to 3D printing was described in detail by Grab et al. [7]. 

#### 2.2. Setup of the training environment 

**Figure 2:**
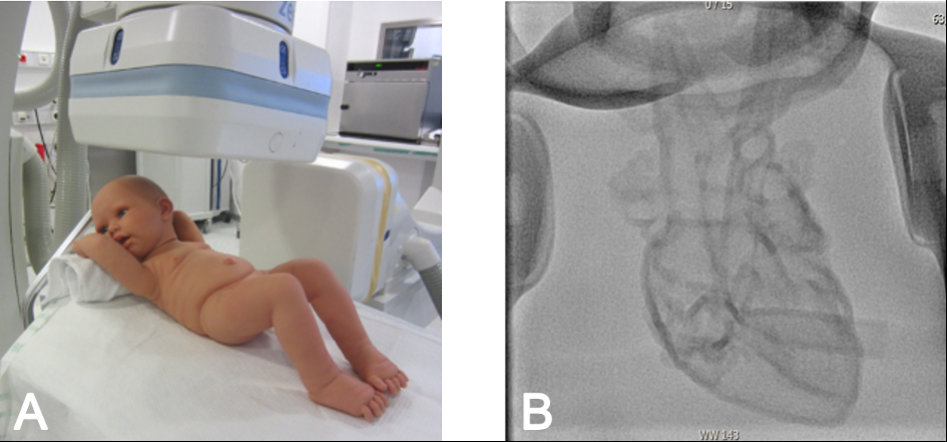
Baby doll with 3D-printed heart model inside the chest A) The doll is positioned on the catherization table like a real patient. B) The 3D-printed heart model of a healthy heart inside the doll can be seen under anterior-posterior fluoroscopy.

To create a realistic training environment the 3D-printed model of a healthy heart was inserted into a life-size plastic baby doll at the beginning of the hands-on training and placed in a realistic position on the CC table (see figure 2 (Fig. 2), section A). The next step included radiological imaging for better understanding and visualization of the topographical anatomy in the different projection directions (see figure 2 (Fig. 2), section B). The 3D-printed models of different CHDs were positioned on the CC table according to the actual position and location of the heart in the body.

#### 2.3. Structure of the CC training modules 

The training courses were designed for students, residents, and experienced pediatric cardiologists. The individual courses took place in the cath lab of the hospital and lasted about seven hours. The senior staff of the Department of Pediatric Cardiology and Pediatric Intensive Care at the LMU Klinikum Großhadern, provided instructions and support acting as supervisor for the participants. For participants without experience, a theoretical teaching unit was provided beforehand to explain the structure of a CC laboratory as an introduction to the principles of CC interventions. Based on a script developed especially for the courses, the basics of cardiac examinations as well as the physiology and hemodynamics of the healthy heart and the CHDs were internalized together before continuing the hands-on training in the cardiac laboratory of the clinic. 

After the participants familiarized their selves with the equipment including the movable table, the X-ray tube and the CC devices, e.g., the airlock of the introducer sheath, the guide wire handling and the different types of catheters and balloons, the practical part of the CC course followed. The exercise in the CC laboratory was always carried out considering radiation protection through appropriate protective clothing and distance to the spectators.

The correct positioning of the doll on the CC table and adequate sterile draping were practiced first. The next step was to attach the airlock to a tube leading to the 3D heart. Subsequently, the handling of the wire, lock and catheter was internalized in several steps.

**Figure 3:**
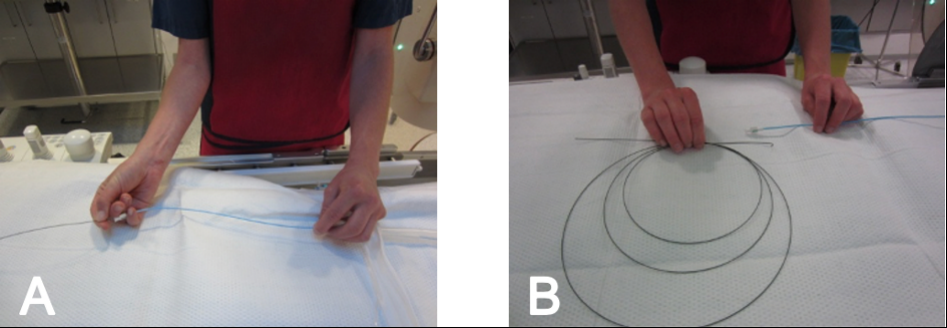
Demonstration of the correct handling of the guide wire A) It was demonstrated how the guide wire is inserted into the catheter and moved forward using one hand. B) It was demonstrated how the long wire can be folded into loops when outside of the catheter to ensure sterile handling.

For example, one exercise included insertion of the guide wire through the catheter (see figure 3 (Fig. 3), section A) and looping it correctly after finally removing it again (see figure 3 (Fig. 3), section B). The eleven participants practiced the individual steps of CC in groups of two using the 3D heart models. Each team was individually supported by the supervisor.

**Figure 4:**
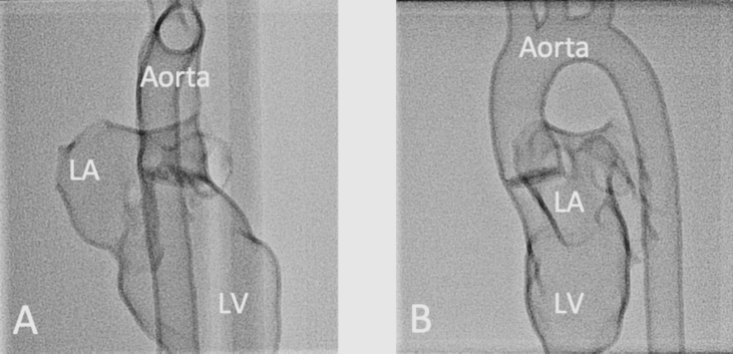
Influence of the projection levels on the anatomic representation during fluoroscopy of a 3D-printed left heart with physiological anatomy in two planes (Aorta = aortic arch, LV = left ventricle, LA = left atrium) A) Representation of a 3D-printed heart model in anterior-posterior fluoroscopy. B) Representation of a 3D-printed heart model in lateral fluoroscopy.

The first exercise was performed on a 3D-printed heart model of the left heart with physiological cardiac anatomy. For this purpose, it was scanned in the anterior-posterior (see figure 4 (Fig. 4), section A) and lateral (see figure 4 (Fig. 4), section B) planes to demonstrate the importance of adequate visualization. The aim was to retrogradely probe the left heart via the descending aorta. Similarly, an exercise was performed on a model with physiologically normal anatomy of the right heart. The participants also learned the intracardiac measurement of pressure curves, saturation, and flow during these exercises. Particular attention was paid to so-called “wire-skills”, such as the technique of changing the catheter and the control of the guide wire and the catheter.

**Figure 5:**
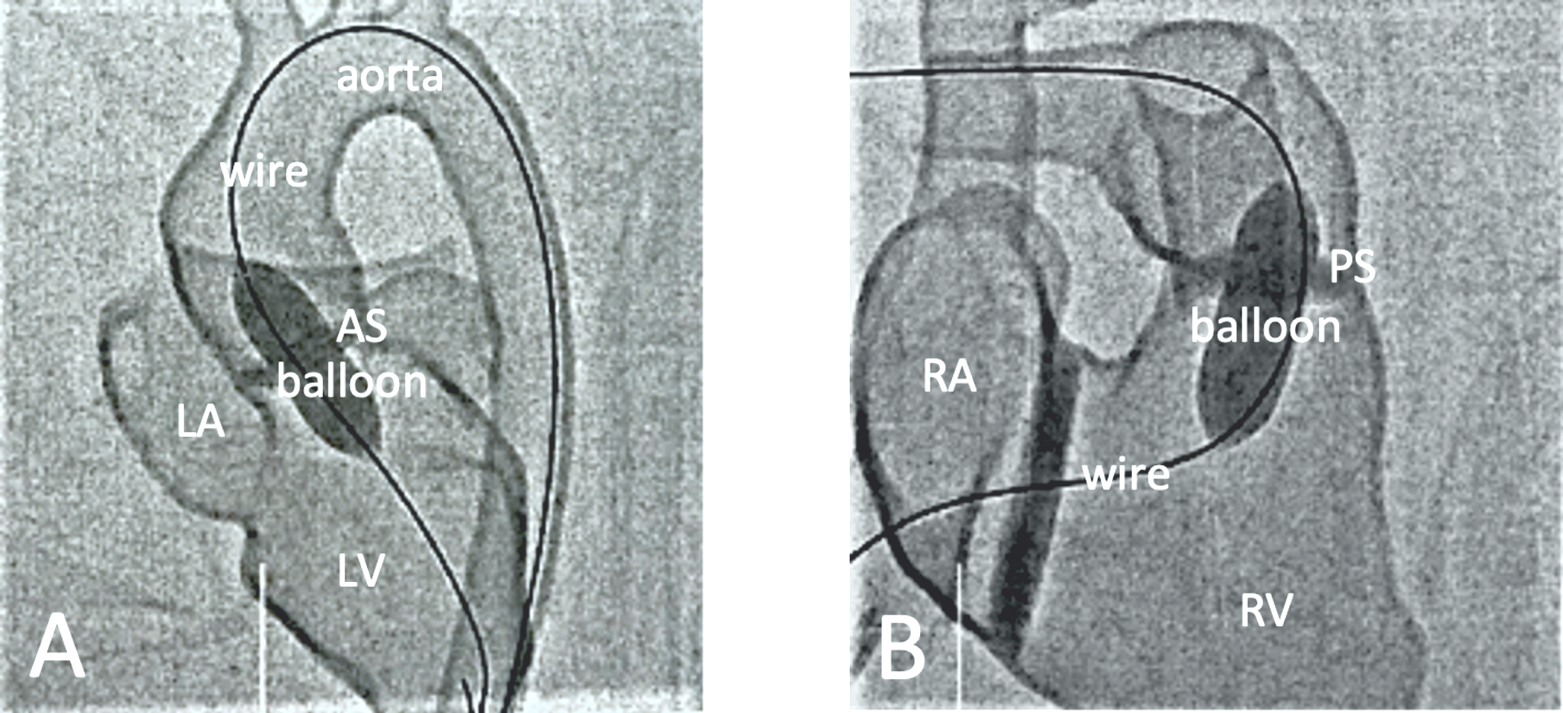
Fluoroscopic documentation of a balloon dilatation of a valvular stenosis within the 3D-printed heart model A) Balloon dilatation of a valvular aortic stenosis. The inflated balloon is positioned at the level of the aortic valve. The long guide wire is inserted via the descending aorta with its tip lying in the left ventricle. B) Balloon dilatation of a valvular pulmonary stenosis. The inflated balloon is positioned at the level of the pulmonary valve. The long guide wire is inserted via the vena cava inferior through the right atrium into the right ventricle with its tip lying in the right pulmonary artery. (Aorta = aortic arch, AS = aortic stenosis, PS = pulmonary stenosis, LV = left ventricle, LA = left atrium, RV = right ventricle, RA = right atrium).

Afterwards, the steps for interventional therapy of the most common CHDs were demonstrated. Special attention was given to the functioning of a balloon catheter and the associated indeflator, as well as practicing the preparation of the balloons and in- and deflation (see figure 5 (Fig. 5)). Umbrellas (occluders) are used to close the ASD. In the case of a PDA, shields or metal coils can be used. Both closure implants have a special technique for correct positioning and release by the guiding catheter. After the demonstration, the participants practiced the intervention steps and the positioning of the implants on the models. 

#### 2.4. Evaluation 

To evaluate the newly developed 3D models and the simulation training itself, the personal progress and the subjective evaluation of the participants were surveyed. The entire statistical analysis was done using Microsoft Excel.

For example, the exercise on the model with and without PS focused on the time needed by the participants to advance the guide wire and catheter from the inferior vena cava via the right atrium and ventricle into the pulmonary artery. Each participant performed this exercise twice on the anatomically correct model. Afterwards, the same exercise was performed on the model with PS, whereby the level of difficulty increased by the stenosis of the valve. These three runs were counted as one training session. The fluoroscopy times (FL-time) of these three runs were compared among each other. A reduction in FL-time during the training session corresponded to the personal progress of the participant. The significance was tested using the sign-test with a significance level of 0.05.

At the end of each course, an anonymous questionnaire, specifically developed for the evaluation of the 3D models and the simulation training was filled in by each participant. The evaluation was based on a Likert scale with values ranging from one (strongly agree) to five (strongly disagree). A total of ten items was queried. The suitability of the 3D-printed heart models was assessed using four items for learning theoretical basics including diagnostic procedures and catheter intervention steps. Another four items were used to assess the suitability for learning the following learning contents: independent practice and repetition, better understanding of cardiac anatomy, understanding of the procedure of CC interventions, and learning how to use the catheterization devices. The assessment of the potential benefits of hands-on training, such as the opportunity for practice and patient safety, were considered based on the participants' experiences. The ratings “strongly agree” and “rather agree” were summarized to indicate agreement, and “rather disagree” and “strongly disagree” to indicate disagreement with the method. Finally, the individual feedback of the participants was evaluated qualitatively by free text comments.

## 3. Results

Since the development of the 3D models in 2018, four courses have taken place, two courses at the LMU hospital and two courses at the General Hospital of the City of Vienna. 19 medical students and doctors participated in total. Among them there were 14 participants with no experience in the CC laboratory, two residents in training with moderate experience and three fully trained pediatric cardiologists with a high level of experience in the CC laboratory. 

### 3.1. Fluoroscopy time

The FL-times presented here show the results of a course at the LMU hospital with eleven participants without knowledge and experience in the CC laboratory, as the assessment of the acquired skills in this homogeneous group provides the best representation of the training effect. 

The sequentially measured FL-times of the participants in three exercise rounds on the physiological (1^st^ and 2^nd^ round) and PS model (3rd round) were compared. The participants (n=11) showed a significant difference in the measured FL-times during the three repetitions (sign test: *p<0.05, ***p<0.001).

**Figure 6:**
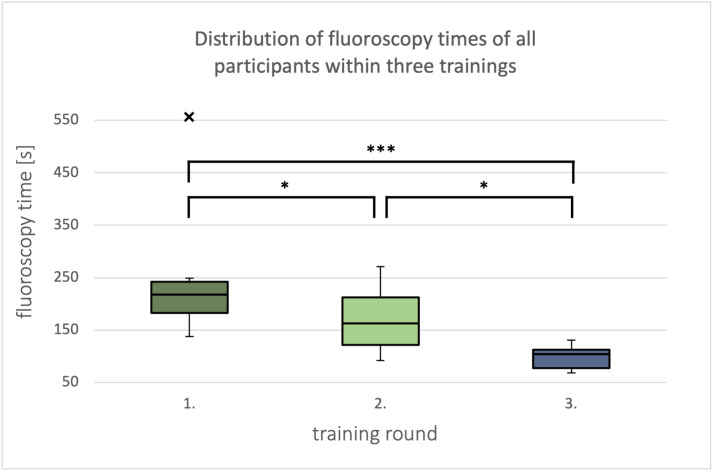
Development of the median distribution of fluoroscopy times of all participants (n=11) in three training rounds The median fluoroscopy time could be reduced from 218 seconds in the beginning to 104 seconds at the end of the third training round. Attention needs to be paid to the fact that the 1st and 2nd round were performed on the model of a healthy heart whereas the same steps were performed on a model with valvular pulmonary stenosis in the 3rd round. Thus, the last round contained an additional difficulty because the wire and catheter had to be steered past the obstacle of the pulmonary valve stenosis. Nevertheless, a significant difference, i.e., decrease of fluoroscopy time, among these three rounds could be shown. (Sign test: *p<0,05, ***p<0,001).

Figure 6 (Fig. 6) shows the distribution of the participants’ (n=11) FL-times over three practice rounds. The median FL-time was reduced from 218 seconds in the first to 104 seconds in the third round despite the increase in task complexity using the PS model in the third round.

#### 3.2. Questionnaires

The results presented in the following include the submitted questionnaires of all participants of all courses conducted so far. Overall, the exercise on the 3D-printed heart models was rated very positively by all participants (n=19, 84.2% “very good”, 15.8% “good”). Most of the participants agreed that the cardiac intervention steps could be internalized using 3D-printed models. 

##### 3.2.1. Assessment issues 

**Figure 7:**
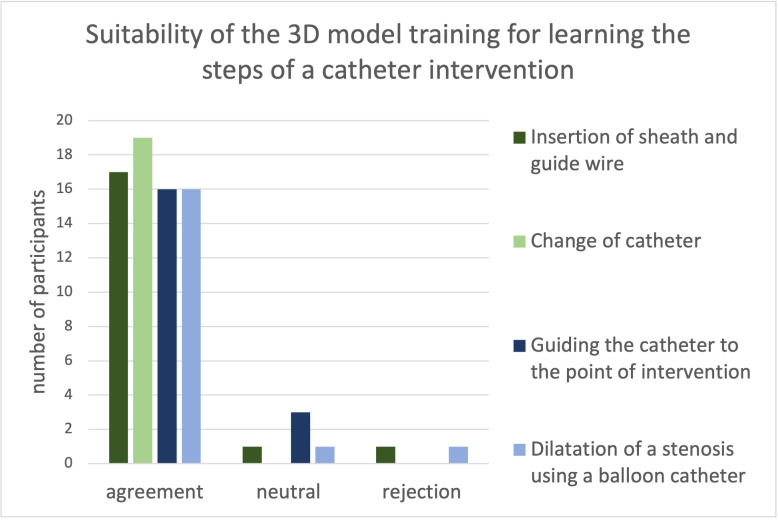
Responses of all participants (n=19) divided into approval and rejection regarding the suitability of 3D-printed heart models to learn the steps of a catheter intervention Altogether the data showed a broad acceptance regarding the suitability of learning the steps of cardiac catheterization using 3D-printed heart models. All participants (n=19) agreed that changing a catheter can be trained on the models. Only few participants felt that the heart models were less suitable to learn how to insert a sheath and wire and to dilate a stenosis (n=18).

The following diagrams show the items from the questionnaire listed under 2.4 Evaluation. In the evaluation of learning specific steps of the cardiac examination on the 3D-printed heart model especially the correct handling of the wire and catheter exchange was assessed as a suitable learning unit by all participants within the framework of the course. The insertion of the sheath and guide wire as well as the dilatation of stenoses were rated as less suitable or neutral or rejected by individual participants (see figure 7 (Fig. 7)).

**Figure 8:**
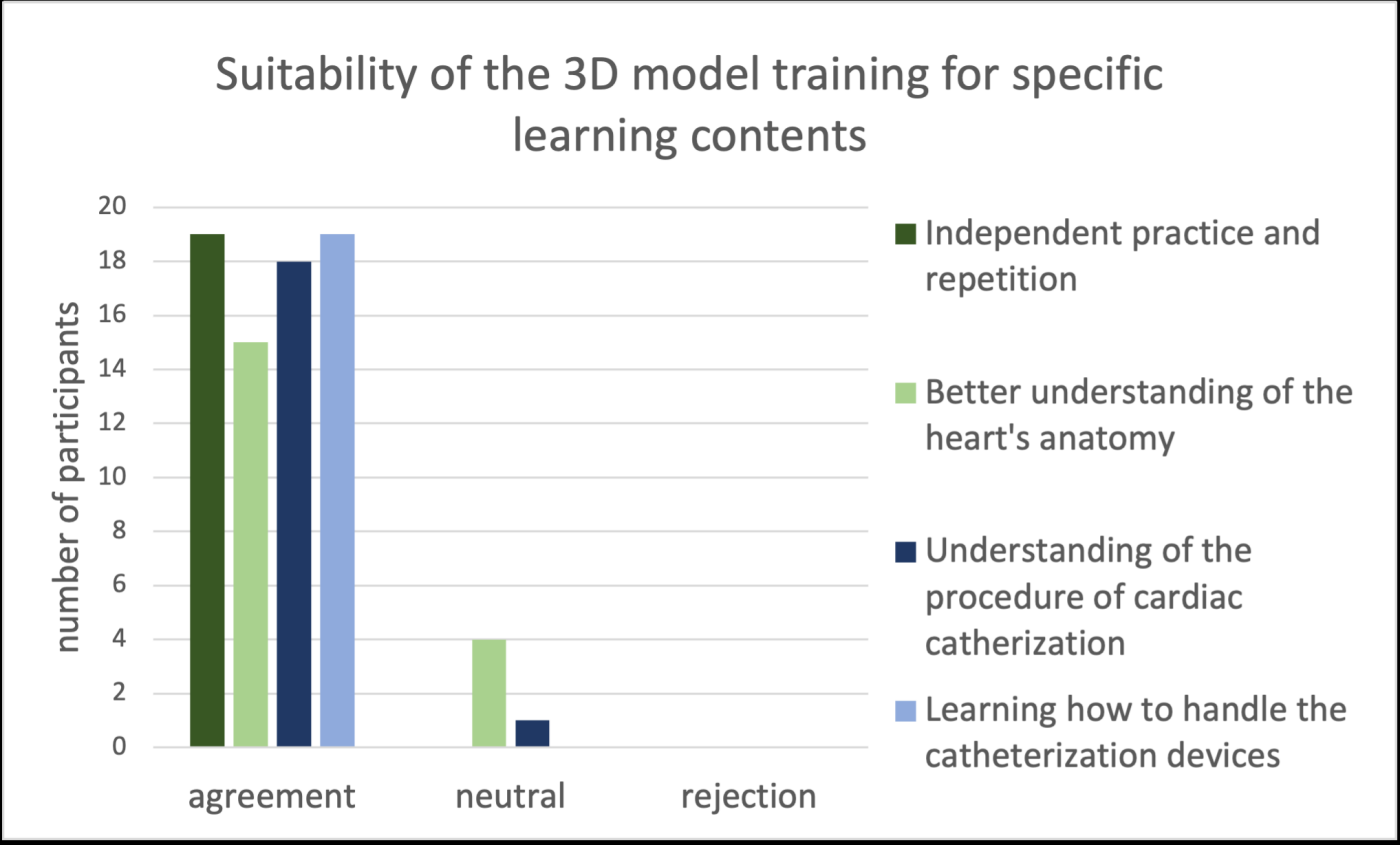
Responses of all participants (n=19) divided into approval and rejection regarding the suitability for certain learning contents All participants (n=19) agreed that it is possible to practice and repeat independently as well as to learn the handling of catheterization devices. Only few participants felt that the training on 3D-printed heart models was less suited for understanding the anatomy of the heart and the procedure of catheter interventions. The were no rejections.

All participants agreed that the models were well suited for independent practice and for learning how to use the catheterization devices. There were no negative stands in this block of questions (see figure 8 (Fig. 8)). 

**Figure 9:**
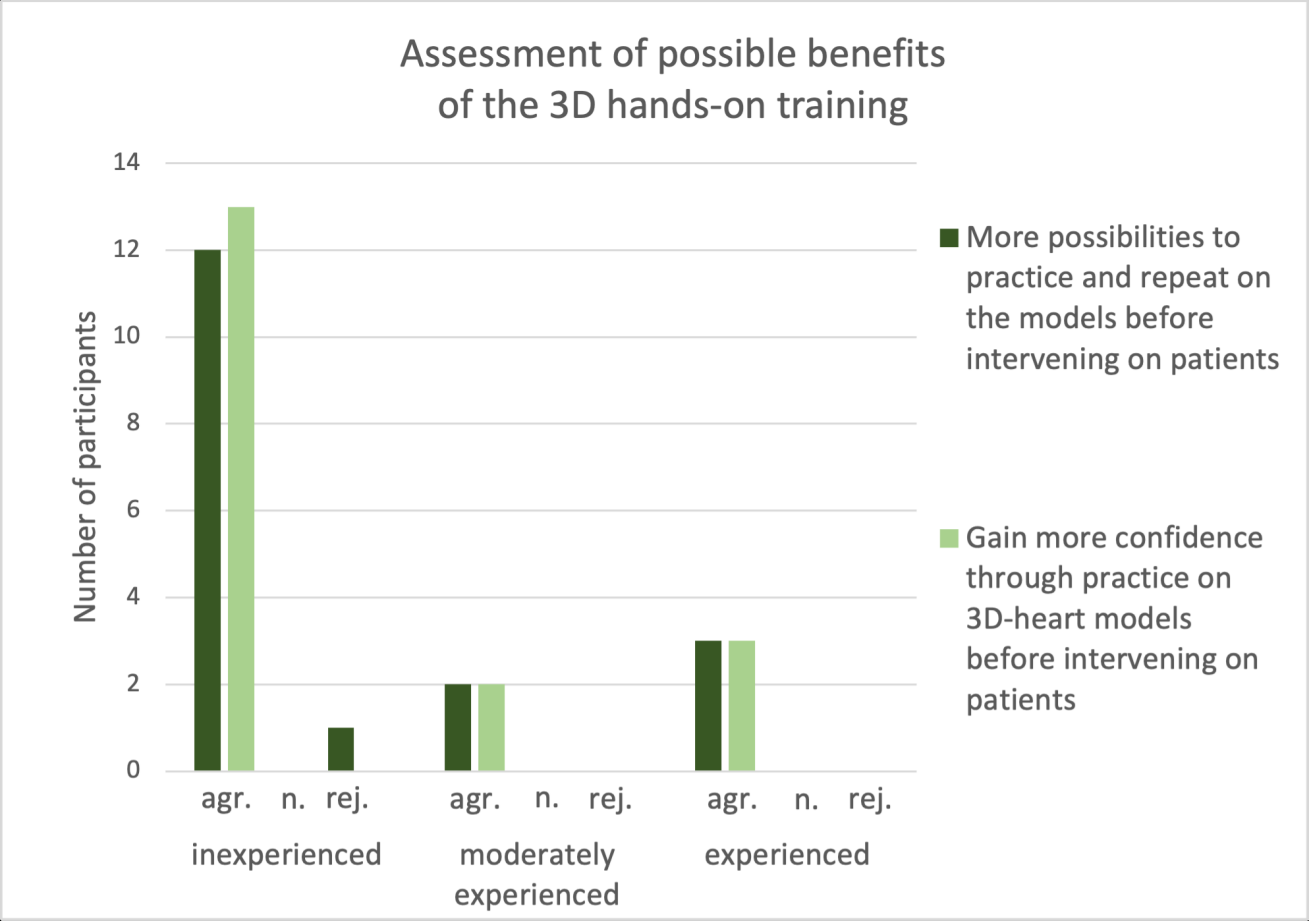
Assessment of possible benefits of the 3D hands-on training The responses were divided by level of experience (inexperienced (n=14, one abstention), moderately experienced (n=2), experienced (n=3)). For the assessment of possible benefits of the 3D hands-on training the participants’ level of experience was considered. Regardless of the level of experience all participants wished to have more possibilities to use this new method of training. All participants also agreed that the practice on 3D-printed heart models could lead to more confidence when intervening on patients.

For a summarizing and final assessment of the hands-on training the participants were divided according to their experience in the CC laboratory. Participants from all experience levels (n=18) stated that there should be more opportunities to practice on 3D models in the future to achieve higher safety in patient interventions (see figure 9 (Fig. 9)). 

##### 3.2.2. Free text comments 

The evaluation of the free text comments enabled an assessment of the subjective evaluation by the participants (n=19). Four participants emphasized the closeness to reality of the models estimating the possibility of practicing “without fear of destroying something”. Five participants particularly mentioned the simple and clear explanation of the exercises. Five participants also liked the structure of the courses, especially the linkage between theory and practice. The high proportion of practical exercises was noted positively by six participants. Participants of the largest group, which included eleven people, suggested a smaller group size (n=8) and better time management (n=5). Four comments offered criticism on the 3D-printed models. It was noted that the ASD and PDA were difficult to reach with the catheter inside the 3D-printed models due to the given anatomical structure. In addition, the silicone rubber sometimes caused resistance due to friction between the catheter and the silicone model.

## 4. Discussion

In pediatrics, the establishment of simulation training has become increasingly important over the last two decades [8]. Nowadays, simulation-based training is a component of many pediatric residency programs worldwide, but predominant in the area of resuscitation and trauma management. Simulation training has proven to be particularly useful in consolidating procedural skills [9].

Throughout the studies of medicine, models and simulations are used throughout the training period to support teaching. In physiology, simulation models support the understanding and visualization of processes and interrelationships [10]. Since the amendment of the medical licensing regulations in 2002, learning practical skills has been an essential task during medical studies [11], [12]. For this purpose, training facilities, so-called “skill labs”, have been established to teach practical, basic medical skills in small groups under standardized conditions [13]. The use of 3D-printed models in anatomy courses for medical students or medical staff does not only improve interest, but also medical training [14], [15].

3D-printed models have been used in other areas of medicine, such as pediatric cardiac surgery [16], neurosurgery [17], otorhinolaryngology [18] and colorectal surgery [19] and have been established as valuable planning and simulation aid. On the one hand, they support the basic understanding of anatomical features and cardiac anomalies [20]. On the other hand, they support doctor-patient communication through tangible visualization, which is particularly advantageous in the preoperative setting and for individual surgical planning in CHDs [21], [22], [23], [24], [25].

The 3D-printed models are a cheaper and more available alternative compared to other clinical simulators [26]. In addition, they can be produced in different scales, variances and as often as desired. 

So far, the models have hardly been used for practical training by pediatric cardiologists. However, based on the results obtained during this study, it has been proven that 3D-printed models are highly suitable for training purposes in this field. 

Within the scope of the course, it has been shown that the FL-time could be reduced by repeating a consistent exercise sequence several times. Further simulation courses using 3D-printed models and longitudinal data collection are necessary to assess the sustainability of the learning effect. Heidbuchel, Chambers and Katz were able to show in their respective studies that a reduction in radiation exposure could already be achieved in interventional (adult) cardiology through targeted training and special courses [27], [28], [29]. In the long term, a significant reduction in radiation exposure for patients can be expected through regular basic and advanced training. 

The evaluation of the questionnaires showed that the participants were very satisfied overall with the training on the 3D-printed models. The participants from all levels of experience agreed that they would profit from such training opportunities in the future confirming the acceptance of this type of training. Training on a model provides space for making mistakes and for learning how to deal with difficult situations without directly exposing patients to risk [30]. Burkhardt and Ziv both emphasized the importance of such simulation possibilities in their studies [30], [31]. 

Regarding higher patient safety, the cardiologist’s confidence in the execution of the intervention steps is also very important. A study from 2002 was able to show that virtual simulation training of laparoscopic interventions resulted in shorter operation times and significantly better performance of the surgeons [32]. This can most likely also be achieved by practicing in the CC laboratory on the 3D-printed heart model.

The fact that simulation training in pediatric cardiology is suitable to achieve the same results, was shown by the evaluation of the participants of all experience levels. Everyone has the chance to learn at their own pace gaining confidence in the individual intervention steps. The number and intensity of training sessions needed to offer a lasting effect is still unclear. Weininger explains the importance of more intensive training at the beginning of the learning curve pointing out that repetition units are still necessary to maintain the training effect [33]. In addition, simulation training and clinical work with patients could complement each other. However, hands-on training is linked to the availability of the CC laboratory and is associated with a higher radiation exposure of the trainee, which is why sufficient radiation protection must be ensured [27], [28], [29].

Based on the feedback of the participants, the heart models are continuously re-evaluated and revised. For example, the friction between the devices and the model was reduced in the short term by using silicone spray. In the long term, however, a fluid-filled, pulsatile 3D model is planned. This will enable further exercise possibilities such as intracardiac pressure measurements and angiographies using contrast agent. Visual and haptic feedback will also be improved. Models of other heart defects are already planned for training additional CC techniques. Due to the use of the models in the hospital’s own CC laboratory and the resulting local flexibility it is possible to offer such training courses worldwide. An example is a clinic partnership project funded by GIZ between the Department of Pediatric Cardiology and Pediatric Intensive Care at the LMU Hospital Großhadern and the Ayder Referral Hospital in the city of Mekelle, Ethiopia. As part of the project, Ethiopian doctors without experience in catheterization are trained in the CC laboratory using the 3D-printed models. The progress of the practical skills is documented and evaluated during the project.

## 5. Conclusion

It was possible to develop a realistic training module for learning diagnostic and interventional cardiac examinations for therapy of the most common CHDs. The use of 3D-printed heart models of different sizes, corresponding to the age groups of patients, contributes to the resemblance of reality of the simulation. The effectiveness and practicability of the hands-on training was evaluated showing that practicing on the 3D-printed heart models was accepted and positively evaluated as a type of training by all participants. This training setup seems to be extremely beneficial for optimizing an individual learning curve offering the possibility to train in a risk-free setting, to gain confidence and, thus, to increase patient safety. For the future, this new kind of training offers numerous opportunities for education and training on the job, as well as the potential to be established as an integral part of interventional (pediatric) cardiology. 

## Notes

The authors Barbara S. Brunner and Alisa Thierij share the first authorship.

The results of this paper are part of the MD thesis of the two first authors and C. Hopfner.

## Competing interests

The authors declare that they have no competing interests.
